# Statistical and Biological Gene-Lifestyle Interactions of *MC4R* and *FTO* with Diet and Physical Activity on Obesity: New Effects on Alcohol Consumption

**DOI:** 10.1371/journal.pone.0052344

**Published:** 2012-12-21

**Authors:** Dolores Corella, Carolina Ortega-Azorín, Jose V. Sorlí, M. Isabel Covas, Paula Carrasco, Jordi Salas-Salvadó, Miguel Ángel Martínez-González, Fernando Arós, José Lapetra, Lluís Serra-Majem, Rosa Lamuela-Raventos, Enrique Gómez-Gracia, Miquel Fiol, Xavier Pintó, Emilio Ros, Amelia Martí, Oscar Coltell, Jose M. Ordovás, Ramon Estruch

**Affiliations:** 1 Department of Preventive Medicine and Public Health, School of Medicine, University of Valencia, Valencia, Spain; 2 CIBER Fisiopatología de la Obesidad y Nutrición, Instituto de Salud Carlos III, Madrid, Spain; 3 Cardiovascular Epidemiology Unit, Municipal Institut for Medical Research (IMIM), Barcelona, Spain; 4 Human Nutrition Unit, Faculty of Medicine, IISPV, University Rovira i Virgili, Reus, Spain; 5 Department of Preventive Medicine and Public Health, School of Medicine-Clínica, University of Navarra, Pamplona, Spain; 6 Department of Cardiology, Hospital Txagorritxu, Vitoria, Spain; 7 Department of Family Medicine, Primary Care Division of Sevilla, San Pablo Health Center, Sevilla, Spain; 8 Department of Clinical Sciences, University of Las Palmas de Gran Canaria, Las Palmas de Gran Canaria, Spain; 9 Department of Nutrition and Food Science, Pharmacy School, University of Barcelona, Barcelona, Spain; 10 Department of Epidemiology, School of Medicine, University of Malaga, Málaga, Spain; 11 University Institute for Health Sciences Investigation, Hospital Son Dureta, Palma de Mallorca, Spain; 12 Lipids and Vascular Risk Unit, Internal Medicine, Hospital Universitario de Bellvitge, Hospitalet de Llobregat, Barcelona, Spain; 13 Lipid Clinic, Endocrinology and Nutrition Service, Institut d’Investigacions Biomèdiques August Pi Sunyer (IDIBAPS), Hospital Clinic, Barcelona, Spain; 14 Department of Nutrition, Food Science, Physiology and Toxicology, University of Navarra, Pamplona, Spain; 15 Department of Computing Languages and Systems, University Jaume I, Castellon, Spain; 16 Nutrition and Genomics Laboratory, JM-USDA Human Nutrition Research Center on Aging at Tufts University, Boston, Massachusetts, United States of America; 17 Department of Cardiovascular Epidemiology and Population Genetics, Centro Nacional de Investigaciones Cardiovasculares (CNIC), Madrid, Spain; 18 IMDEA Alimentación, Madrid, Spain; 19 Department of Internal Medicine, Hospital Clinic, IDIBAPS, Barcelona, Spain; McGill University, Canada

## Abstract

**Background:**

Fat mass and obesity (*FTO*) and melanocortin-4 receptor (*MC4R*) and are relevant genes associated with obesity. This could be through food intake, but results are contradictory. Modulation by diet or other lifestyle factors is also not well understood.

**Objective:**

To investigate whether *MC4R* and *FTO* associations with body-weight are modulated by diet and physical activity (PA), and to study their association with alcohol and food intake.

**Methods:**

Adherence to Mediterranean diet (AdMedDiet) and physical activity (PA) were assessed by validated questionnaires in 7,052 high cardiovascular risk subjects. *MC4R* rs17782313 and *FTO* rs9939609 were determined. Independent and joint associations (aggregate genetic score) as well as statistical and biological gene-lifestyle interactions were analyzed.

**Results:**

FTO rs9939609 was associated with higher body mass index (BMI), waist circumference (WC) and obesity (P<0.05 for all). A similar, but not significant trend was found for MC4R rs17782313. Their additive effects (aggregate score) were significant and we observed a 7% per-allele increase of being obese (OR = 1.07; 95%CI 1.01–1.13). We found relevant statistical interactions (P<0.05) with PA. So, in active individuals, the associations with higher BMI, WC or obesity were not detected. A biological (non-statistical) interaction between AdMedDiet and rs9939609 and the aggregate score was found. Greater AdMedDiet in individuals carrying 4 or 3-risk alleles counterbalanced their genetic predisposition, exhibiting similar BMI (P = 0.502) than individuals with no risk alleles and lower AdMedDiet. They also had lower BMI (P = 0.021) than their counterparts with low AdMedDiet. We did not find any consistent association with energy or macronutrients, but found a novel association between these polymorphisms and lower alcohol consumption in variant-allele carriers (B+/−SE: −0.57+/−0.16 g/d per-score-allele; P = 0.001).

**Conclusion:**

Statistical and biological interactions with PA and diet modulate the effects of *FTO* and *MC4R* polymorphisms on obesity. The novel association with alcohol consumption seems independent of their effects on BMI.

## Introduction

Early prediction of obesity risk will be facilitated by the identification of the main genes and their relevant genetic variants. However, the next step involving more successful obesity prevention and therapy will better achieve after learning how the phenotypic expression of the relevant genetic variants are modulated by pertinent lifestyle factors such as dietary intake and physical activity in order to reduce the manifestation of obesity in genetically predisposed individuals. Outstanding among the many genes associated with obesity is the fat mass and obesity gene (*FTO*). It was the first locus discovered to be associated with BMI and obesity risk [1]. One year after, a meta-analysis of several genome wide association studies GWAs [2], established that the second most important locus associated with BMI was the melanocortin-4 receptor gene (*MC4R*). Previous studies had already identified various rare mutations in the *MC4R* as the commonest cause of monogenic forms of early-onset obesity [3], [4]. Of the various polymorphisms found both in the *FTO* and in the MC4R loci, the most significant are rs9939609 and rs17782313, respectively [1], [2], [4]. The minor allele of the rs9939609 (C>A), has been consistently associated with higher BMI and obesity risk [1], [5]–[8]. In a meta-analysis of population-based studies [1], the per-A allele odds ratio (OR) for obesity was 1.31 [95% confidence interval (CI): 1.23–1.39]. Although the minor allele of the rs17782313 (T>C), has also been significantly associated with greater BMI [2], [4], [9]–[11], its relevance in terms of the magnitude of the association is considered smaller than that of *FTO* polymorphism, being the per-allele OR for obesity of 1.12 (95% CI: 1.08–1.16) in the initial meta-analysis [2]. A few studies have also jointly analyzed the *FTO* and *MC4R* polymorphisms, describing their additive effects on obesity related variables [1], [9], [12], [13]. Thus in a European population, Cauchi et al [13] found that, compared to participants carrying neither *FTO* nor *MC4R* risk allele, subjects with three or four risk alleles had a 1.8-fold increased obesity risk.

The mechanism by which these polymorphisms may be associated with greater BMI is unknown, but some studies on animals have suggested that for both of them it could be through a higher food intake [14]–[16]. However, human studies investigating the associations of these polymorphisms with dietary intake are scarce and contradictory [17]–[26]. Although initial studies [17]–[19] have reported that *FTO* polymorphisms were associated with higher fat and energy intake in carriers of the variant alleles, other studies have not been able to replicate these associations [20]–[23]. Studies on the *MC4R* locus are few and also divergent [23]–[26], requiring more research. Likewise, whereas several studies have found an important statistical interaction with physical activity for the *FTO* locus, so that greater physical activity attenuates the increasing effects of the A-allele on BMI [27]–[29], for the *MC4R* polymorphism there are hardly any studies that have analyzed this interaction [30], [31].

Although recently several studies analyzed the possible modulation of the effects of the *FTO* polymorphisms on BMI through diet, the results are still differing [22], [32]–[35]. This, and the lack of studies focused on gene-diet interactions of the *MC4R* polymorphism, make it necessary to carry out new research on these gene-diet interactions considering both loci jointly. Moreover, although the vast majority of studies have focused on statistical interactions [36], [37], we also have the so-called “biological interactions” in which environmental factors could modify genetic susceptibility without the interaction term in the mathematical model being statistically significant [36]–[39]. Hence our aims were: 1) To analyze the associations of the *MC4R* rs17782313 and *FTO* rs9939609 polymorphisms with BMI and their modulation by physical activity and diet, including the overall adherence to the Mediterranean diet (MedDiet) pattern; 2) To study whether these polymorphisms have an influence on food and alcohol intake in a high cardiovascular risk population.

## Methods

### Subjects

We included 7,052 participants (3,008 men and 4,044 women) from the PREDIMED (PREvención con DIeta MEDiterránea) study from whom DNA was isolated, the *FTO* polymorphism (rs9939609) determined, and who had valid data for the main clinical and lifestyle variables analyzed at baseline. In 7,019 of them, the *MC4R* rs17782313 polymorphism was successfully determined. The PREDIMED study is a multi-center clinical trial (controlled-trials.com number, ISRCTN35739639) aimed at assessing the effects of the MedDiet on the primary prevention of cardiovascular disease [40]. The 7,052 participants analyzed here did not differ in the main characteristics from those of the total cohort (n = 7,447). Details of the PREDIMED Study have been fully described elsewhere [41]. Briefly, from October 2003 potential high cardiovascular risk subjects were selected by physicians in Primary Care Centers. Eligible subjects were community-dwelling people (55–80 years of age for men; 60–80 years of age for women) who fulfilled at least one of two criteria: Type 2 diabetes; 3 or more cardiovascular risk factors (hypertension, dyslipidemia, body mass index [BMI] ≥25 kg/m2, current smoking, or a family history of premature cardiovascular disease). The specific cut-off points for these eligibility criteria have been previously described [41]. Type 2 diabetes was diagnosed according to American Diabetes Association criteria [42]. Exclusion criteria included a personal history of cardiovascular disease, any severe chronic illness, and drug or alcohol addiction [41]. Participants were randomly assigned to three interventions [41]. In this study we analyzed data at baseline. The Institutional Review Board/Ethics Committee of each participating center (University of Valencia, Valencia; Municipal Institut for Medical Research, Barcelona; University Rovira i Virgili, Reus; University of Navarra, Pamplona; Hospital Primary Care Division of Sevilla, San Pablo Health Center, Sevilla; University of Las Palmas de Gran Canaria, Las Palmas de Gran Canaria; University of Barcelona, Barcelona; University of Malaga; Málaga; Hospital Son Dureta, Palma de Mallorca; Hospital Universitario de Bellvitge, Hospitalet de Llobregat, Barcelona and Institut d’Investigacions Biomèdiques August Pi Sunyer (IDIBAPS), Hospital Clinic, Barcelona, Spain) approved the study protocol. All participants provided written informed consent.

### Demographic, Clinical, Anthropometric and Dietary Measurements

The baseline examination included assessment of standard cardiovascular risk factors, medication use, socio-demographic factors and lifestyle variables, as previously detailed [41]. Food intake and alcoholic beverage consumption, including wine, beer, and spirits, were assessed through a validated semi-quantitative 137-item food frequency questionnaire [43]. Energy and nutrient intake were calculated from Spanish food composition tables [44]. We calculated alcohol intake (g/d) for each individual on the basis of the type and amount of alcoholic beverages by multiplying the amount of the beverage (mL) by the respective grade (% alcohol) and the constant 0.80 to transform alcohol volumes into weight. In addition, three groups of alcohol consumption were defined according to the reported daily intake of alcohol: no intake (0 g/d), moderate intake (<26.4 g/d for men and <13.2 g/d for women), and high intake (26.4 g/d for men and 13.2 g/d for women). These gram amounts correspond to 1 drink/d for women and 2 drinks/d for men [45].

The baseline examination also included the administration of a validated 14-item questionnaire indicating the degree of adherence to the traditional MedDiet [46]. This screener consists of 14 questions on food consumption frequency and on food intake habits considered characteristic of the Spanish MedDiet. Each question was scored 0 or 1. The final score ranged from 0 to 14. The greater the score obtained from the questionnaire, the greater the adherence to the MedDiet. Dichotomous variables of adherence to the MedDiet and nutrient intake were created using as cut-off points the sample means. Physical activity was estimated by the Minnesota Leisure Time Physical Activity Questionnaire, validated for its use in Spanish subjects [47], [48].

Weight and height were measured with calibrated scales and a wall-mounted stadiometer, respectively. BMI was calculated as weight in kilograms divided by the square of height in meters.

### DNA Extraction and Genotyping

At baseline, blood samples were obtained from each participant after an overnight fast and were frozen at −80°C and shipped to central laboratories for analyses [35]. Genomic DNA was extracted from buffy-coat with the MagNaPure LC DNA Isolation Kit (Roche Diagnostics, Mannheim, Germany). The *MC4R* rs17782313 and *FTO* rs9939609 polymorphisms were genotyped on a 7900HT Sequence Detection System (Applied Biosystems, FosterCity, CA, USA) using fluorescent allelic discrimination TaqManTM assays. The calling rate for both polymorphisms was >95%. For quality control purposes, 5% of samples were randomly selected samples and genotyped a second time. There were no discrepancies between the two results.

### Statistical Analyses

Chi-square tests were used to test differences between observed and expected genotype frequencies, assuming Hardy–Weinberg equilibrium, and to test differences in percentages. Genetic variables were tested using co-dominant models of the polymorphisms individually. In addition, an additive genetic score was created from the *FTO* and the *MC4R* polymorphism so that the presence of each of variant allele for each polymorphism was scored as one point according to the work of Cauchi et al [13]. The range of values of this aggregate score variable was from 0 (homozygous subjects for non-variant alleles) to 4 points (homozygous subjects for the variant alleles both at the *FTO* and *MC4R* genes). In addition, due to the low prevalence of homozygous subjects for the variant alleles, a grouped score variable was created grouping the categories of 3 and 4 points as previously described [13]. We used t and ANOVA tests to compare crude means of obesity indexes across genetic categories. Multivariate adjustments for comparisons of continuous variables were carried out by generalized linear models. Models were first adjusted for age, sex, center and diabetes. Additional adjustments for energy intake, physical activity, adherence to the MedDiet or BMI were also carried out as indicated. In addition to the categorical analysis, the *FTO* and the *MC4R* polymorphism were considered in the co-dominant model as linear terms coded 0, 1, or 2 (additive effect), depending of the number of variant alleles with the homozygote wild-type coded as 0. The regression coefficient (B) and the 95% confidence interval (CI) was estimated for each polymorphism. This coefficient indicates the change of Y for each variant allele of the tested polymorphism. Likewise, the aggregate score was also considered as a linear term coded as 0, 1, 2, 3, or 4 depending on the number of variant alleles. The corresponding B and 95%CI for the aggregate score was also estimated in the unadjusted and adjusted regression models.

Dichotomous variables for dietary variables and physical activity were created using as cut-off the sample means. Alcohol intake was square-root transformed for statistical testing. Logistic regression methods were also used to estimate the associations of the *MC4R* rs17782313 and *FTO* rs9939609 polymorphisms to predict obesity prevalence and to adjust for confounders as indicated. In addition to the categorical analysis, the *FTO* and the *MC4R* polymorphism were considered in the co-dominant model as linear terms coded 0, 1, or 2 (allele dosage effect), depending of the number of variant alleles with the homozygote wild-type coded as 0. The regression coefficient (B) and the 95% confidence interval (CI) was estimated for each polymorphism. This coefficient indicates the change of Y for each variant allele of the tested polymorphism. Likewise, the aggregate score was also considered as a linear term coded as 0, 1, 2, 3, or 4 depending on the number of variant alleles. The corresponding B and 95%CI for the aggregate score were also estimated in the unadjusted and adjusted regression models.

The homogeneity of the effects by sex was also statistically tested using the likelihood ratio test. To test the interaction between the *MC4R* rs17782313, the *FTO* rs9939609 polymorphisms or their score variable and physical activity, adherence to MedDiet or the other dietary variables, separated multivariate regression models including the corresponding main effects and interaction terms in addition to the potential confounders, were fitted. The likelihood ratio test was used to obtain the P values for interactions. Stratified analyses were also carried out. Statistical analyses were performed with the SPSS package, version 15.0 (SPSS, Chicago, IL). All tests were two-tailed and P values <0.05 were considered statistically significant.

Power calculations were performed using QUANTO software, v.1.2.4 [49] and Power and Sample Size Calculation (biostat.mc.vanderbilt.edu/wiki/Main/PowerSampleSize). Initial sample size estimations were undertaken assuming an allele frequency for the minor *FTO* and MC4r alleles of 0.43 and of 0.23, respectively and the parameters from the meta-analysis by Frayling et al [1] for the *FTO* (a 31% per-allele increase in the odds of being obese) and the parameters from the meta-analysis by Loos et al [2] for the MC4R (a 12% per-allele increase in the odds of being obese), respectively. To detect these reported effects our sample size (n = 7,019) had a power higher that 80% (alpha-level = 0.05). Our study was adequately powered (>80%) to detect as statistically significant a 7% per-allele increase in the OR for obesity for the additive effects of both polymorphism in the aggregate score in the population as a whole. The power to detect a statistically significant gene-environment interaction effect higher than OR 1.20 in the logistic regression model (using a dichotomous environmental variable based on the population mean) was >95% for the *FTO* (additive effects) and >80% for the *MC4R.* However, as the gene-interaction effect of the MC4R was 1.10, the power to detect this interaction as statistically significant was <50%. For the aggregate score, the power to detect the above described additive gene-environment interaction effects in determining obesity was >80%. For power calculation in the linear regression analysis, we considered the magnitude of the gene-environment interaction, and according to Luan et al [50] an interaction was considered to be relevant in magnitude when the ratio of the two regression slopes [B1 for the genetic variable as additive in the first versus the second (B2) stratum was at least 2]. With a sample size of 7019 individuals our study has a power>80% to detect this interaction effect for the different outcomes analyzed. Finally, this number of study subjects allowed us a power>90% to detect as statistically significant mean differences of dietary intake by genotype based on previously reported significant studies [17]–[19], [24].

## Results

**Table 1** shows demographic, clinical, lifestyle and genetic characteristics of the 7,052 participants in the PREDIMED study at baseline depending on the *FTO* rs9939609 and the *MC4R* rs17782313 polymorphisms. Because of the selection criteria, the prevalence of obesity was high (46.8%). The mean (±SD) of adherence to the MedDiet was 9±2 points on the scale of 0 to 14 points in the whole populations. Genotype frequencies did not deviate from Hardy-Weinberg equilibrium expectations for either polymorphisms (P = 0.709 for the *FTO* rs9939609 and P = 0.637 for the *MC4R* rs17782313). The aggregate score of the two polymorphisms had a prevalence of 20.6% for zero points (homozygous subjects for non-variant alleles); 40.9% for 1 point (subjects with one variant allele either at *FTO* or *MC4R*; 29.2% for 2 points (subjects with two variant alleles), 8.5% for 3 points (subjects with 3 variant alleles) and 0.8% for 4 points (homozygous subjects for the variant alleles both at the *FTO* and *MC4R* genes). As the number of subjects with a score of 4 points was very low, a new score-grouped variable was created grouping the categories of 3 and 4 points.

**Table 1:** Demographic, clinical, lifestyle and genetic characteristics of the study participants according to the *FTO* and the *MC4R* polymorphisms at baseline*.

	*FTO* rs9939609 (n = 7,052)						*MC4R* rs17782313 (n = 7,019)					
	TT (n = 2329)		TA (n = 3434)		(AA = 1289)		TT (n = 4336)		TC (n = 2553)		(CC = 330)	
Age (years)	67.0	(6.2)	67.1	(6.3)	66.6	(6.1)	66.9	(6.2)	67.1	(6.2)	66.7	(6.4)
Adherence to the Mediterranean diet (points)	8.6	(1.9)	8.7	(2.0)	8.7	(1.9)	8.7	(2.0)	8.7	(2.0)	8.6	(1.9)
Energy intake (kcal/d)	2288.2	(616.1)	2277.3	(606.4)	2250.8	(593.9)	2274.3	(607.1)	2279.2	(611.4)	2309.4	(593.0)
Physical activity** (kcal/d)	228.7	(234.6)	235.2	(241.7)	225.8	(245.3)	231.4	(239.5)	235.1	(242.6)	217.5	(240.4)
Men (%)	42.6		42.7		42.7		42.9		42.8		40.3	
Current smokers (%)	14.2		13.9		14.3		13.6		14.9		14.8	
Diabetes (%)	47.3		49.0		50.1		48.5		48.8		49.1	
Hypertension (%)	82.7		82.8		82.1		82.3		83.4		82.1	
Dyslipidemia(%)	72.9		71.8		72.0		72.4		71.9		71.2	
Obesity (%)	45.1		46.6		50***		46.4		46.9		49.4	
*FTO* rs9939609: TT (%)							62.0		32.9		5.1	
*FTO* rs9939609: TA (%)							61.7		33.7		4.6	
*FTO* rs9939609: AA (%)							61.5		34.3		4.2	
*MC4R* rs17782313: TT (%)	33.4		48.5		18.1							
*MC4R* rs17782313: TC (%)	32.5		48.8		18.6							
*MC4R* rs17782313: CC (%)	35.2		48.3		16.5							

* : Values are means and standard deviations (SD) for continuous variables or percentages for categorical variables.

** : Leisure time physical activity.

*** : Statistically significat differences (P<0.05) among genotypes.

### Association between the FTO rs9939609 and MC4R rs17782313 Polymorphisms and Anthropometric Variables at Baseline

On analyzing the population as a whole we found a statistically significant association between the *FTO* polymorphism and body-weight, BMI and waist circumference so that carriers of the variant allele (A) presented statistically significant higher mean values than homozygous subjects for the major allele (**Table 2**). These associations were statistically significant in the unadjusted analysis and remained statistically significant even after multivariate control for sex, age, center, diabetes, total energy intake and physical activity (fully adjusted model). The regression coefficient (B), assuming additive effects of the variant allele of *FTO* were also statistically significant for body-weight, BMI and waist circumference. For BMI, we observed an increase of 0.17 kg/m^2^ (95%CI: 0.05–0.30) for each additional variant allele. This increase, although statistically significant was not clinically relevant. On stratifying by sex (data not shown), this association was of greater magnitude in women than in men. However, the interaction terms between the *FTO* polymorphism and sex did not reach statistical significance for any of the anthropometric measurements analyzed (P>0.05 for all of them). With regard to the *MC4R* polymorphism, its association with BMI and waist circumference was of lower magnitude than for *FTO*. Although a trend was observed for greater values of BMI and waist circumference in homozygous subjects for the variant allele (C-allele), statistical significance was not reached in the total population (Table 2). However, we obtained statistically significant results for body-weight (0.60 kg per-variant allele; 95% CI: 0.17–1.03) and for height (0.3 cm per-variant allele; 95%CI: 0.01–0.05) in the adjusted analysis. Although subjects carrying variant alleles for the *MC4R* polymorphism were also significantly taller (P = 0.031) than their counterparts, the magnitude of the effect was very small. On analyzing the effects of these polymorphisms on obesity we found similar results (**Table 2**). The *FTO* polymorphism was significantly associated with a higher prevalence of obesity (a 10% per-allele increase in the odds of being obese; 95%CI: 2%–17%). Homozygous subjects with the variant *FTO* allele conclusively demonstrated a 20% higher risk for obesity (model adjusted for sex, age, center, diabetes, total energy intake, and physical activity; OR 1.20, 95% CI 1.04–1.38). There was a suggestion that homozygous subjects with the variant *M4CR* allele had a 12% higher risk for obesity but this finding was not conclusive (OR: 1.12, 95% CI 0.89–1.41). Although our study was adequately powered to detect the effect reported by Loos et al [2] for the *MC4R*, as finally the magnitude of the effect in our population was lower than that initially expected, our study was unpowered (<80%) to detect as statistically significant the small effect found for the *MC4R* in the whole population.

**Table 2:** Association between the *FTO* and the MC4R polymorphisms and anthropometric measures. Crude means, odds ratio (OR) and unadjusted and adjusted* regression coefficients (B).

			*FTO* or *MC4R* genotypes							
	11		12		22			Crude	Adjusted*	
	Mean	SD	Mean	SD	Mean	SD	P^1^	B (95% CI)	B (95% CI)	P^2^
			***FTO*** ** rs9939609**							
	TT (n = 2329)		TA (n = 3434)		(AA = 1289)					
Weight (kg)	76.4	(11.8)	76.7	(11.9)	77.7	(12.3)	0.008	0.57 (0.18, 0.96)	0.47 (0.11, 0.82)	0.011
Height (cm)	160.0	(0.1)	160.0	(0.1)	160.0	(0.1)	0.739	0.001 (−0.003, 0.005)	0.000 (−0.002, 0.002)	0.881
BMI (kg/m^2^)	29.8	(3.8)	29.9	(3.8)	30.3	(4.1)	0.006	0.20 (0.06, 0.34)	0.17 (0.05, 0.30)	0.007
Waist (cm)	99.9	(10.8)	100.5	(10.4)	101.2	(10.5)	0.005	0.59 (0.24, 0.94)	0.53 (0.19, 0.87)	0.003
Obesity, OR (95% CI)	1 (ref.)		1.06 (0.96–1.18)		1.22 (1.07–1.40)		0.016	1.10 (1.03–1.17)	1.10 (1.02–1.17)	0.014
			***MC4R*** **rs17782313**							
	TT (n = 4336)		TC (n = 2553)		(CC = 330)					
Weight (kg)	76.5	(11.8)	77.1	(12.1)	77.8	(12.2)	0.079	0.56 (0.09, 1.03)	0.60 (0.17, 1.03)	0.008
Heigh (cm)	159.9	(0.1)	160.0	(0.1)	160.1	(0.1)	0.481	0.003 (−0.001, 0.007)	0.003 (0.001, 0.005)	0.031
BMI (kg/m^2^)	29.9	(3.8)	30.1	(3.8)	30.4	(4.1)	0.098	0.14 (−0.02, 0.30)	0.13 (−0.02, 0.28)	0.119
Waist (cm)	100.3	(10.4)	100.7	(10.8)	100.5	(11.1)	0.309	0.28 (−0.15, 0.71)	0.30 (−0.11, 0.71)	0.186
Obesity, OR (95% CI)	1 (ref.)		1.02 (0.91–1.41)		1.13 (0.91–1.41)		0.561	1.04 (0.96–1.13)	1.03 (0.95–1.12)	0.454

Values are means and standard deviations (SD), odds ratio (OR) and 95% confidence intervals (CI) or regression coefficients (B) and 95% CI.

1 : Unadjusted P values for the polymorphisms for the comparison of means or OR as categorical (three categories).

B: Regression coefficient per-variant allele effects (genotypes coded as 0, 1 and 2 according to the number of minor alleles; additive effects).

* : Models adjusted for sex, age, center, diabetes, total energy intake and physical activity.

2 : P-values obtained in the adjusted regression models considering additive effects for the polymorphisms.

**Figure 1:**
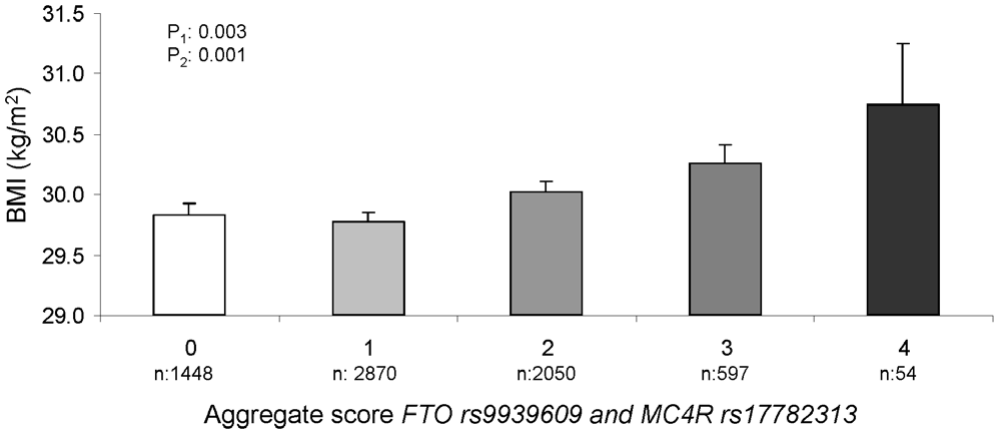
Association between the aggregate genetic score of the *FTO* rs9939609 and *MC4R* rs17782313 and body mass index (BMI). Multivariate adjusted means. Error bars: SE of means. 0 points (non-variant alleles); 1 point (one variant allele either at *FTO* or *MC4R*; 2 points (two variant alleles), 3 points (3 variant alleles) and 4 points (4 variant alleles). P^1^: unadjusted P-value for the comparison of means; P^2^: adjusted for sex, age, center, diabetes, energy intake and physical activity.

On creating the aggregate score variable for the two polymorphisms, we observed that the higher the number of risk alleles, the higher the average BMI (**Figure 1**) and waist circumference (not shown), reaching statistically significant results (with a increase of B±SE: 0.16±0.05 kg/m^2^; P = 0.001, per-variant allele for BMI and B±SE: 0.43±0.13 cm; P = 0.001, per-variant allele for waist circumference in the adjusted model). Likewise, on considering the aggregate score variable a statistically significant association with obesity was found. In the fully adjusted model (sex, age, center, diabetes, total energy intake and physical activity, each variant allele (*FTO* or *MC4R*) of the additive score increased the odds of obesity by 7% (OR = 1.07; 95%CI: 1.01–1.13) for the continuous aggregate score variable).

### Gene-physical Activity Interaction between the FTO rs9939609 and MC4R rs17782313 Polymorphisms on Determining Anthropometric Variables

**Figure 2:**
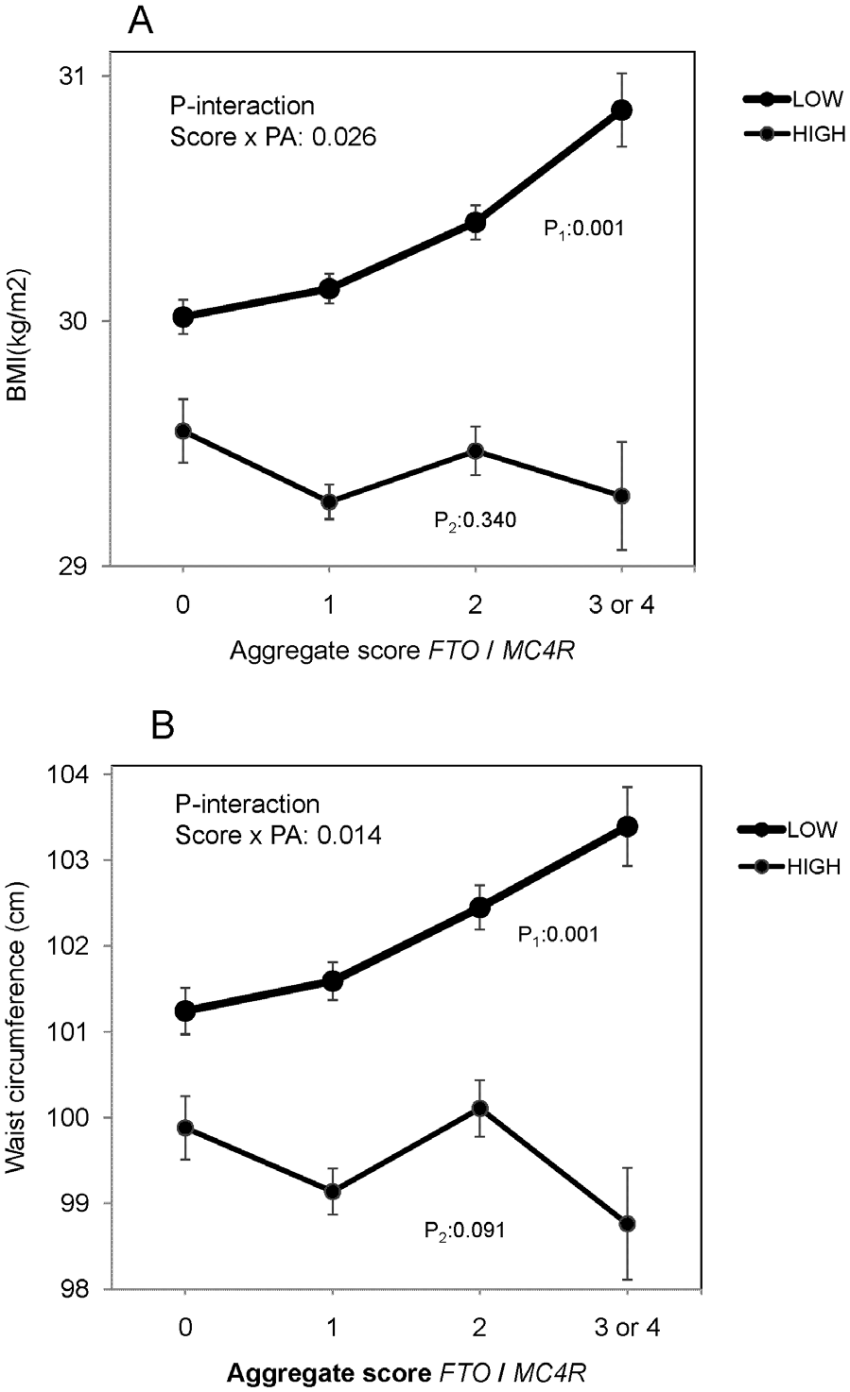
Statistical interaction between the aggregate score (grouped) of the *FTO* rs9939609 and *MC4R* rs17782313 and physical activity on BMI (A) or waist circumference (B). Adjusted means (n = 7,019) of BMI or waist circumference depending on the aggregate score (0, 1, 2, 3 or 4 variant alleles) according to the strata of physical activity (below and above 230 kcal/d). Means were adjusted for sex, age, center, diabetes and total energy intake. P values for the interaction terms were multivariate adjusted. In the stratified analysis by physical activity, P values for mean comparisons between genotypes in the low (P_1_) and high strata (P_2_) were multivariate adjusted. Error bars: SE of means.

We found a relevant statistically significant interaction between the *FTO* rs9939609 polymorphism and leisure time physical activity on BMI (P-interaction = 0.010) that remained statistically significant (P-interaction = 0.015) in the multivariate adjusted model (**Figure S1A**). Thus, in subjects with high leisure time physical activity (higher than the sample mean, 230 kcal/d) we did not detect a statistical significant association between the *FTO* polymorphism and BMI (mean±SE: 29.5±0.1 kg/m^2^ in TT, 29.3±0.1 kg/m*^2^* in TA and 29.5±0.2 kg/m*^2^* in AA; P = 0.689). However, when physical activity was low, we did find a significant association between the *FTO* rs9939609 variant allele and higher BMI (mean±SE: 29.9±0.1 kg/m^2^ in TT, 30.3±0.1 kg/m^2^ in TA and 30.7±0.2 kg/m^2^ in AA; P = 0.001). Likewise, a similar statistically significant interaction (P-interaction = 0.005 in the fully adjusted model) was observed for waist circumference (**Figure S1B**). Thus, when physical activity was high (higher than the sample mean) we did not detect a statistically significant association between the *FTO* polymorphism and waist circumference (mean±SE: 99.5±0.4 cm in TT, 99.6±0.3 cm in TA and 99.4±0.5 cm in AA; P = 0.737). However, when physical activity was low, we did find a significant association between the *FTO* rs9939609 variant allele and higher waist circumference (mean±SE: 100.2±0.4 cm in TT, 101.1±0.3 cm in TA and 102.3±0.5 cm in AA; P<0.001). Despite not finding a similar interaction between the *MC4R* polymorphism and physical activity in determining anthropometric variables (P-interaction = 0.485 for BMI and P-interaction = 0.151 for waist circumference), we found a statistically significant interaction between physical activity and the aggregate score of both polymorphisms on both BMI (P-interaction = 0.026) (**Figure 2A**) and waist circumference (P-interaction = 0.014) (**Figure 2B**).

On considering obesity (**Table 3**) we also found a statistically significant interaction between physical activity and the *FTO* polymorphism (P-interaction = 0.007 for linear allelic effects), that was more significant when the aggregate genetic score variable was analyzed (P-interaction = 0.005 for lineal allelic effects). This interaction effect was clinically relevant as the magnitude of the association was different between the strata. Thus, when physical activity was low, the *FTO* polymorphism and the aggregate score were significantly associated with higher obesity risk in carriers of the variant allele (a 17% per-allele increase; 95%CI: 7–27 and a 12% per-allele increase; 95%CI: 5–20 in the OR of obesity, respectively). However, when physical activity was high, the *FTO* polymorphism or the aggregate score were not associated with higher odds of obesity (P = 0.956 and P = 0.376, respectively), suggesting that a high level of physical activity is likely to overcome the detrimental effects of genetic predisposition to obesity even in a high-risk population. This observation was not due to a lack of power to detect statistically significant differences in the stratum of high physical activity for which similar effects than for the low strata were detectable, but the magnitude of the OR was very low and even in the opposite direction (lower than 1).

**Table 3:** Association of the *FTO, MC4R* and the combined score (*FTO* and *MC4R* polymorphisms) with obesity. Stratified multivariate* logistic regression analysis according to physical activity.

		Physical activity (PA)									
		Low (<230 kcal/d)				High (> = 230 kcal/d))				P^2^ for interaction	
		OR		95% CI		OR		95% CI		Genotype×PA	
***FTO*** ** rs9939609** (n = 7,052)											
TT		1.00		(reference)		1.00		(reference)			
TA		1.12		(0.98–1.29)		0.95		(0.79–1.14)			
AA		1.39		(1.16–1.65)		0.92		(0.73–1.16)			
		P^1^ = 0.001				P^1^ = 0.743					
*Variant allele effects* **											
(Per-A allele)		1.17		(1.07–1.27)		0.96		(0.85–1.07)		0.007	
**MC4R rs17782313** (n = 7,019)										0.519	
TT		1.00		(reference)		1.00		(reference)			
TC		1.05		(0.92–1.20)		0.94		(0.79–1.11)			
CC		1.19		(0.89–1.58)		0.99		(0.67–1.48)			
		P^1^ = 0.412				P^1^ = 0.758					
*Variant allele effects* **											
(Per-C allele)		1.07		(0.96–1.18)		0.96		(0.78–1.09)		0.251	
**Aggregate score (** ***FTO*** **/** ***MC4R*** **)**										0.008	
TT and TT (0)		1.00		(reference)		1.00		(reference)			
TA or TC (1)		1.06		(0.90–1.25)		0.81		(0.65–1.01)			
TA and TC or AA or CC (2)		1.14		(0.96–1.36)		0.91		(0.72–1.14)			
Otherwise (3 or 4 variants)		1.58		(1.24–2.01)		0.75		(0.54–1.05)			
		P^1^ = 0.001				P^1^ = 0.173					
*Variant allele effects* ***											
(Per-allele increase: 1,2,3,or 4)	1.12		(1.05–1.20)		0.96		(0.87–1.05)		0.005		

* : Models adjusted for sex, age, center, diabetes, total energy intake and leisure time physical activity.

** : For the estimation of the variante allele efects a variable indicating the number of variant alleles (0, 1 or 2) was created for *FTO* and *MC4R.*

*** : For the estimation of the variant allele effect of the aggregate score a variable indicating the number of combined variant alleles (0, 1, 2, 3 or 4) was created.

P^1^: P-value obtained for the global effect of the polymorphism in the multivariate logistic regression models.

P^2^: P-value for the interaction term between physical activity polymorphism and the corresponding polymorphism in the logistic regression model.

CI: Confidence interval.

### Gene-diet Interactions between the FTO rs9939609 and MC4R rs17782313 Polymorphisms in Determining Anthropometric Variables

For the *FTO* polymorphism we did not find statistically significant interactions with the MedDiet on BMI (P-interaction = 0.614) or on waist circumference (P-interaction = 0.229), but the results obtained are compatible with the so-called “biological interaction”. That is to say the *FTO* polymorphism does not present genetic determinism on BMI or waist circumference. Homozygous subjects for the risk-allele may have higher or lower BMI or waist circumference depending on their greater (above the sample mean: 9 points) or lesser adherence to the MedDiet (**Figure S2A and S2B**). Hence, AA subjects (homozygous for the obesity risk allele) with low adherence to the MedDiet had a statistically significant higher BMI than AA individuals with high adherence (mean±SE: 30.5±0.2 kg/m^2^ *vs* 29.7±0.2 kg/m^2^, respectively; P = 0.008 in the adjusted model). Likewise, mean waist circumference was statistically higher in AA subjects with low adherence to the MedDiet in comparison with AA subjects with high adherence (mean±SE: 102.5±0.4 cm *vs* 100.5±0.4 cm, respectively; P = 0.001 in the adjusted model). These effects can be considered clinically relevant in magnitude. Moreover, for waist circumference, higher adherence to the MedDiet significantly reversed the genetic effect. Thus, average waist circumference in AA subjects having high adherence to the MedDiet was significantly lower than average waist circumference in TT subjects with low adherence (mean±SE: 100.4±0.4 cm *vs* 101.5±0.3 cm, respectively; P = 0.033).

**Figure 3:**
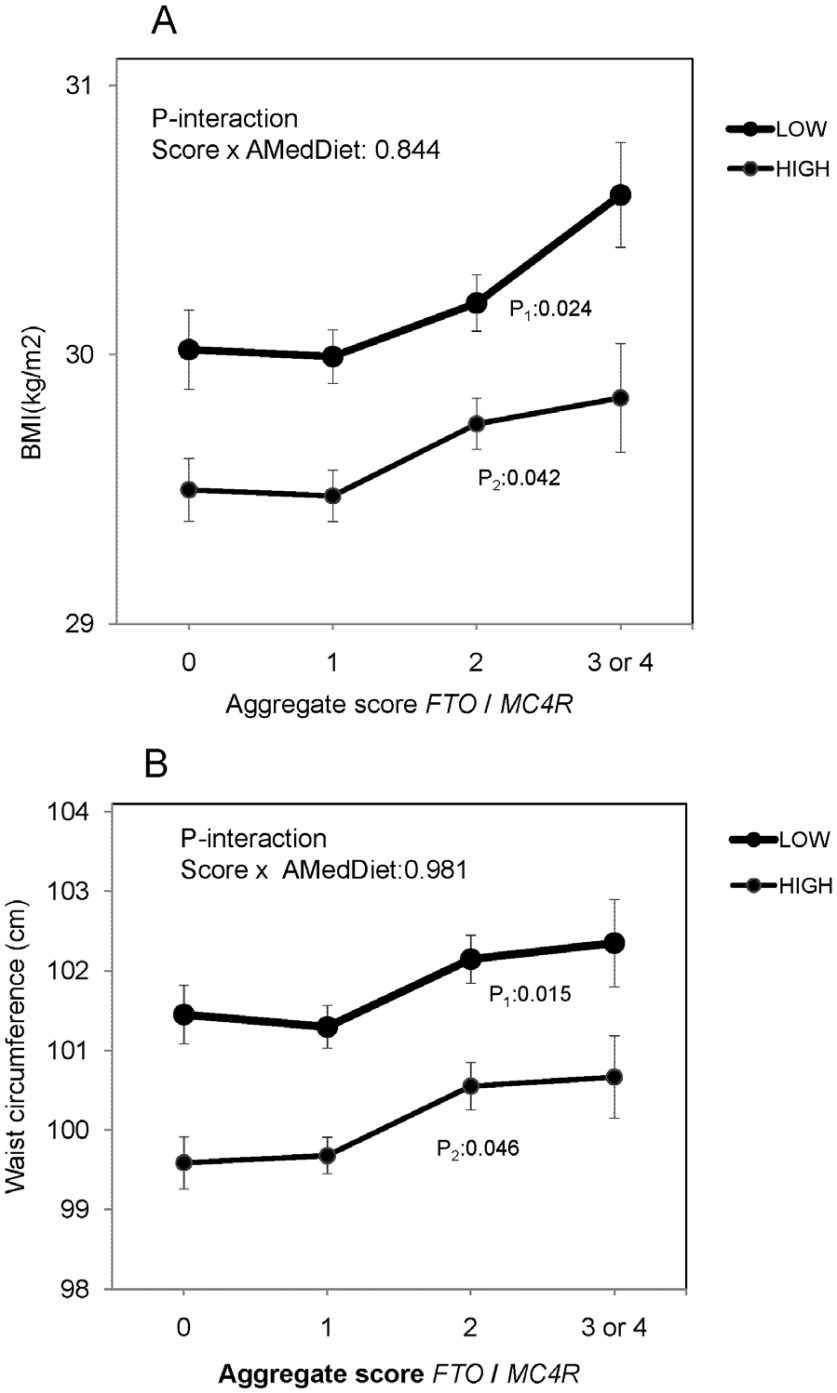
Interaction between the aggregate score (grouped) of the *FTO* rs9939609 and *MC4R* rs17782313 and adherence to the Mediterranean Diet (AdMedDiet) on BMI (A) or waist circumference (B). Adjusted means (n = 7,052) according and to the strata of AdMedDiet (below and above 9 points). Means were adjusted for sex, age, center, diabetes, total energy intake and physical activity. P values for the interaction terms were multivariate adjusted. In the stratified analysis by AdMedDiet, P values for mean comparisons of BMI or waist circumference between genotypes were multivariate adjusted (P_1_ for the low AdMedDiet and P_2_ for high). Error bars: SE of means.

Similar results were observed when analyzing the aggregate score of both polymorphisms on BMI (**Figure 3A**) or waist circumference (**Figure 3B**). Although we did not obtain statistically significant interactions between adherence to the MedDiet and the aggregate score on BMI (P-interaction = 0.844) or on waist circumference (P-interaction = 0.981), we observed a “biological interaction”. Despite we did not find statistically significant differences in the slopes of the aggregate score in the two strata of adherence to MedDiet (B1∶0.19; 95%CI: 0.05–0.34 in the low strata vs B2∶0.16; 95%CI: 0.02–0.29 in the high strata) and the B1/B2 ratio was <1.2 (absence of a statistically significant interaction), we clearly observed that the BMI of individuals with higher genetic risk will depend on their greater o lesser adherence to the MedDiet (“biological interaction”). Carriers of 3 or 4 variant alleles had significantly higher BMI when adherence to the MedDiet was low in comparison with their counterparts having a higher adherence (mean±SE: 30.6±0.2 *vs* 29.8±0.2 kg/m^2^, respectively; P = 0.021). Interestingly, high adherence to the MedDiet in individuals genetically susceptible (carriers of 4 or 3 variant alleles) counterbalanced their genetic predisposition to higher BMI, exhibiting similar average BMI than individuals with no risk alleles and lower adherence to the MedDiet (mean±SE: 29.8±0.2 *vs* 30.0±0.2 kg/m^2^, respectively; P = 0.502). For waist circumference we observed similar results. Carriers of 3 or 4 variant alleles had significantly higher waist circumference than their counterparts when adherence to the MedDiet was low *vs* high (mean±SE: 102.3±0.6 *vs* 100.7±0.5 cm, respectively; P = 0.038).

### Association between the *FTO* rs9939609 *and MC4R* rs17782313 and Alcohol and Food Intake

**Figure 4:**
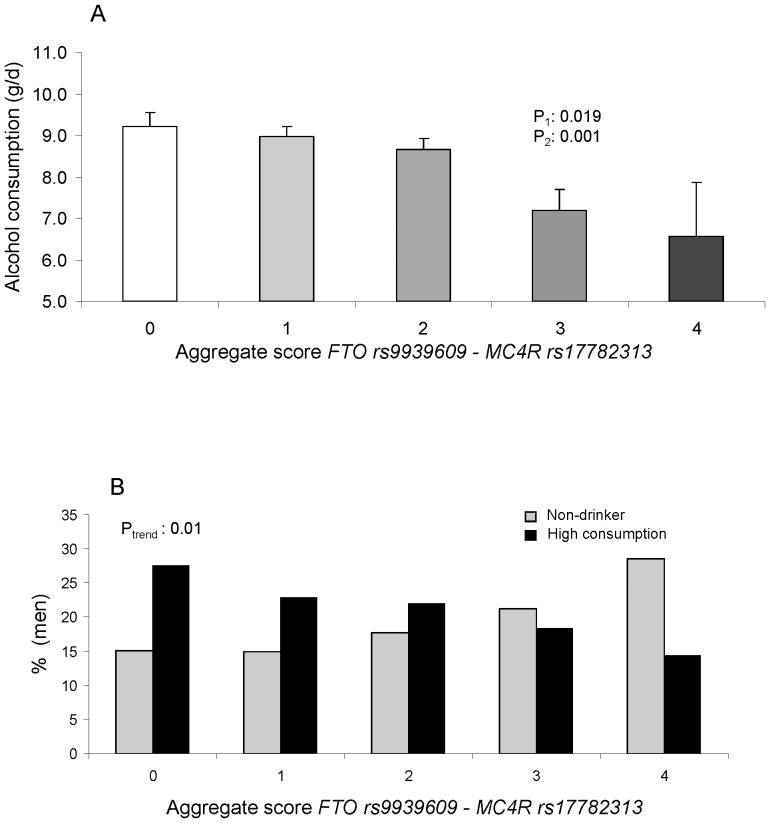
Association between the *FTO* rs9939609 and *MC4R* rs17782313 and alcohol intake in g/d (A) or drinking categories (B). Multivariate adjusted means of alcohol consumption depending on the genetic score of both polymorphisms (A) for the whole population (n = 7,019) or drinking categories (B) in men (n = 2,995). P^1^: unadjusted; P^2^: adjusted for sex, age, center, diabetes, total energy intake and physical activity. Error bars: SE of means.

We did not observe any significant association between the *FTO* or the *MC4R* polymorphisms and food intake (**Table S1**). Interestingly we found statistically significant associations between both polymorphism and total alcohol intake as well as with wine or beer (lower alcohol consumption in subjects carrying the variant alleles). On analyzing the aggregate score variable (**Table 4**), we did not obtain statistically significant associations with energy, macronutrients or food groups, however the association with alcohol consumption was higher and remained statistically significant even after multivariate adjustment for sex, age, center, diabetes, total energy intake, physical activity and adherence to the MedDiet (**Figure 4A**). Moreover, it remained statistically significant even after adjustment for BMI (B = −0.57 g/d per-variant allele; 95%CI: −0.89, −0.25). Per types of alcoholic beverages, we obtained similar results (less consumption in carriers of the variant alleles) for the most consumed alcoholic beverages in this population (wine and beer). Interestingly, when instead of considering alcohol consumption in g/d, we considered subjects as or non-drinkers or drinkers (moderate or high consumption), we also obtained a statistical significant association between the aggregate score and drinking habits. Thus, for the whole population, the prevalence of non-drinkers was higher in subjects with 4 variant alleles in comparison with the prevalence of non-drinkers in subjects with zero variant alleles (43.4% vs 36.5%, respectively; P-trend = 0.01). Likewise, prevalence of subject with high-alcohol consumption was higher in subjects with zero variant alleles (14.5%) than in those with 4 variant alleles (5.7%); P-trend = 0.01). These results were more relevant in men (**Figure 4B**), in which alcohol consumption was higher than in women. Thus, prevalence of non-drinkers was 28.6% in subjects with 4 variant alleles (*vs* 15.1% in non-variant allele carriers), and prevalence of men with high-alcohol consumption was 27% in non-drinkers (*vs* 14.3% in men with 4 variant alleles). These differences can be considered clinically relevant.

**Table 4:** Association between the aggregate score of the *FTO* rs9939609 and the *MC4R* rs17782313 polymorphisms and dietary intake in the whole population. Crude and adjusted analyses.

	Aggregate score *FTO*/*MC4R*									Adjusted*	
	0		1		2		3 or 4		*P* 1	B (95% CI)	*P* 2
	Mean	SD	Mean	SD	Mean	SD	Mean	SD			
Energy intake, kcal*/d*	2292.2	(620.6)	2273.6	(620.6)	2272.7	(611.1)	2273.8	(583.8)	0.775	−6.39 (−21.09, 8.30)	0.394
Total fat, *g/d*	98.8	(31.2)	98.7	(30.2)	99.2	(30.9)	99.2	(29.2)	0.954	−0.03 (−0.75, 0.69)	0.928
SFA, *g/d*	25.3	(9.7)	25.2	(8.9)	25.5	(9.4)	25.4	(9.0)	0.734	0.01 (−0.22, 0.24)	0.934
MUFA, *g/d*	49.0	(16.5)	48.8	(15.9)	49.2	(16.3)	48.8	(15.4)	0.858	−0.08 (−0.44, 0.29)	0.688
PUFA, *g/d*	15.8	(6.9)	15.9	(7.0)	15.9	(7.1)	16.1	(7.0)	0.865	0.05 (−0.13, 0.22)	0.609
Proteins, *g/d*	92.8	(23.8)	92.7	(23.1)	99.2	(23.4)	93.1	(22.9)	0.949	0.01 (−0.58, 0.59)	0.985
Carbohydrates, *g/d*	242.1	(83.3)	238.8	(80.2)	238.2	(81.7)	240.3	(79.7)	0.528	−0.54 (−2.50, 1.46)	0.599
Alcohol, g/d	9.1	(15.5)	8.5	(13.8)	8.2	(14.2)	6.8	(11.7)	**0.009**	−0.57 (−0.89, −0.25)	**0.001**
Wine, g/d	62.3	(117.5)	58.4	(107.4)	55.3	(100.7)	47.2	(92.6)	**0.019**	−3.90 (−6.38, −1.42)	**0.002**
Beer, g/d	49.7	(152.3)	45.2	(137.8)	40.6	(40.6)	33.8	(95.9)	0.051	−5.47 (−8.81, −2.14)	**0.001**
Adherence to MedDiet	8.6	(1.9)	8.7	(2.0)	8.6	(1.9)	8.6	(2.0)	0.169	0.01 (−0.04, 0.05)	0.803
Vegetables, g/d	337.7	(165.5)	338.4	(146.5)	338.6	(148.3)	338.1	(142.7)	0.562	0.46 (−3.24, 4.16)	0.807
Fruits, g/d	373.3	(213.6)	369.0	(204.7)	371.8	(208.3)	379.4	(209.8)	0.103	2.79 (−2.29, 7.88)	0.282
Legumes, g/d	20.9	(15.5)	21.2	(14.4)	20.3	(11.9)	20.9	(13.1)	0.600	−0.19 (−0.53, 0.14)	0.244
Cereals, g/d	148.8	(94.4)	145.6	(87.9)	146.7	(89.9)	143.4	(86.0)	0.569	−0.70 (−2.95, 1.54)	0.540
Dairy products, g/d	374.5	(224.7)	391.2	(227.1)	380.4	(217.1)	386.3	(221.8)	0.870	1.05 (−4.49, 6.59)	0.710
Meats, g/d	134.5	(65.6)	132.0	(59.5)	132.5	(57.1)	133.3	(66.8)	0.764	−0.48 (−1.94, 0.99)	0.521
Fish, g/d	100.9	(49.3)	99.0	(48.2)	100.4	(52.9)	101.1	(66.8)	0.113	0.29 (−0.99, 1.57)	0.662
Pastries, g/d	22.9	(31.4)	22.5	(30.4)	22.7	(30.4)	21.7	(27.5)	0.561	−0.27 (−1.04, 0.50)	0.495
Olive oil, g/d	39.6	(18.2)	39.4	(17.9)	39.4	(18.0)	38.7	(18.4)	0.231	−0.25 (−0.66, 0.16)	0.238
Nuts, g/d	9.9	(13.7)	11.0	(14.8)	10.3	(13.7)	10.3	(13.1)	0.187	0.06 (−0.30, 0.42)	0.761

Values are unadjusted means and standard deviations (SD) or regression coefficients (B) and 95% confidence intervals (CI).

1 : Unadjusted P values for the score variable grouped as four categories.

* : Models adjusted for sex, age, center, diabetes, physical activity and body mass index.

2 : P values for the B (lineal regression coefficient) corresponding to the aggregate score variable as additive (5 levels coded as 0,1,2,3 or 4 according to the number of variant alleles and adjusted for sex, age, field center, diabetes, physical activity and body mass index).

## Discussion

Although the magnitude of the effect in the whole sample was lower that that reported for the general population [1], in this study undertaken in elderly subjects we have found that the *FTO* rs9939609 polymorphism was significantly associated with higher BMI, waist circumference and obesity prevalence, our results coinciding with previous investigations [1], [5]–[11]. However, the *MC4R* rs17782313 polymorphism did not reach the statistical significance as it had a lesser effect in this population and, even though a similar trend was observed. While several studies have reported statistically significant associations between the *MC4R* rs17782313 and BMI or obesity [2], [24], [25], [51], there are also studies that did not find a significant association [9], [52] or found stronger associations with weight than BMI [11] and even studies that have reported an association with higher height [9], [53], [54] and that this association may mask the effects on BMI despite being associated with higher weight. Our results are in agreement with this observation as we found statistically significant differences in weight and height (although very small in magnitude for height).In agreement with other studies [12], [13] we have also found additive effects of these polymorphisms in this population. We observed that the aggregate score of both was associated with higher BMI, waist circumference and obesity prevalence with higher statistical significance and higher magnitude of the effect than the independent polymorphisms.

Interestingly, the magnitude of effects of the *FTO* and *MC4R* polymorphisms were not homogeneous in the whole population. Regarding interactions of these polymorphisms with lifestyle variables, many studies have analyzed the statistical interaction of the *FTO* polymorphism with physical activity obtaining some different results depending on the population [8], [12], [22], [27], [28], [30], [32], [55]. However, a recent meta-analysis including 218,166 adults and 19,268 children [29] concluded that there was a consistent statistically significant interaction between the *FTO* rs9939609 polymorphism and physical activity on obesity risk. Thus, in sedentary subjects the association of the *FTO* polymorphisms with higher BMI or obesity risk was greater in magnitude than in active subjects. In agreement with these results, we also found a consistent statistically significant interaction between the *FTO* rs9939609 polymorphism and leisure time physical activity in determining BMI, waist circumference and obesity prevalence, so that the association between the *FTO*-risk allele and higher BMI or obesity prevalence disappeared in active subjects and was increased in magnitude of the effect and clinical significance in subjects with low physical activity. We did not find any significant interaction between physical activity and the *MC4R* rs17782313 on BMI, waist circumference or obesity, but observed a similar trend. A lack of power to detect the statistical significance was identified for the *MC4R* polymorphism taking into account the lower prevalence of the risk-allele and the lower magnitude of the effect of this polymorphism. However, on considering the aggregate score of the *MC4R* rs17782313 and *FTO* rs9939609, we did observe a statistically significant interaction both for BMI and for obesity. As far as we know this is the first time that a statistically significant interaction between the aggregate score of the *FTO* and the *MC4R* polymorphisms and physical activity on BMI and obesity is reported. Moreover, the magnitude of the effect of this interaction was strong and can be considered as clinically relevant.

With regard to the modulation of the effects of these polymorphisms by diet, there are few studies that have analyzed it in comparison with physical activity, and the consistency level is still low [22], [24], [32]–[35], [56], [57], [58]. Sonestedt et al [32] reported a statistically significant interaction between diet and the *FTO* rs9939609 polymorphism on BMI and found that a high total fat and a low carbohydrate intake boosted the effects of this polymorphism. In another study [22], we concluded that saturated fatty acids were those that presented a more statistically significant and clinically relevant interaction with the *FTO* polymorphism in determining BMI in two American populations. Phillips et al [35] also found a significant interaction with saturated fat intake, replicating our previous findings. However, Vimaleswaran et al [33], analyzing data from five European countries reported no statistically significant interaction between the *FTO* rs9939609 and dietary fat, protein or carbohydrate on BMI. Regarding the *MC4R* rs17782313, the number of studies examining gene-diet interactions on anthropometric variables is very scarce [24]. Qi et al [24] reported no significant interactions between the *MC4R* rs17782313 polymorphism and dietary intake on BMI in The Nurses’ Health Study. In our Mediterranean population we did not find a statistically significant interaction of the *FTO* rs9939609 or the *MC4R* rs17782313 polymorphisms with either total fat or saturated fat intake (data not shown). This is possible due to the fact that we were dealing with a high overall adherence to the MedDiet population in which in general there is a low saturated fat intake and a high MUFA intake from olive oil. We hypothesized that the low or high adherence to the overall MedDiet pattern will be more important than the effect of specific macronutrients. However, when we analyzed the interaction between the adherence to the MedDiet and these polymorphisms on anthropometric variables, we did not find statistically significant gene-diet interactions. The P-values for the interaction terms were non-statistically significant because the effect of the polymorphisms were present (statistically significant) both in the low and the high strata of MedDiet adherence [parallel lines (B1 = B2) in Figure 3]. This was not due to a lack of power to detect the interaction term as statistically significant but to a true absence of heterogeneity of genetic effects between the strata. However, as the MedDiet has also a statistically significant effect on reducing BMI (P<0.05), we observed statistically significant differences between the genotypes depending on the adherence to the MedDiet. We believe that it is important to stress that these observed results are compatible with the so-called “biological gene-diet interaction” [39], [59]. A biological gene-environment interaction situation takes place when there is an environmental factor capable of modifying genetic susceptibility and it is independent of the statistical significance [38], [59]. This biological interaction is opposed to the genetic determinism, in which the genotype completely determines the phenotype. In the genetic determinism situation, if a subject is a carrier of the risk alleles of the *FTO* and the *MC4R* polymorphisms, such genetic susceptibility would irreversibly determine the obesity phenotype, and that genetic influence would be totally independent of lifestyle factors. However, no genetic determinism will occur when dietary modulation exists (in this case higher adherence to the MedDiet) that could modify genetic susceptibility (greater BMI in variant allele carriers) without the interaction term in the mathematical model being statistically significant. Supporting our cross-sectional results, there is an interventional study carried out in a small sub-sample of the PREDIMED participants [51] showing that after 3 years of nutritional intervention with MedDiet, A-allele carriers for the *FTO* rs9939609 had lower body weight gain than TT subjects. Therefore, it seems important to reconsider the concept of gene-diet interaction and not to discard the potential use of other “biological interactions” that could modify genetic susceptibility without the interaction term in the mathematical model being statistically significant [36]–[39], [59].

Regarding dietary intake, we did not find a consistent association of the *FTO* or the *MC4R* polymorphisms with total energy, fat, carbohydrates o protein intake, in agreement with other studies [21]–[23], [34]. However, we do wish to stress the finding of an important association with alcohol consumption. We have observed that carriers of the variant allele, both for the *FTO* rs9939609 and the *MC4R* rs17782313 polymorphisms had statistically significant lower alcohol consumption than wild-type subjects. The magnitude of this effect was higher for the aggregate score and remained statistically significant even after adjustment for BMI. There are no previous studies on humans that have reported an association of the *MC4R* s17782313 polymorphism with alcohol intake although there are several studies on animals which indicate the great importance of the MC4R in determining alcohol intake [60]–[63]. Among them, the work carried out by Navarro et al [61], showing that in mice, when given centrally a melanocortin receptor agonist, melanotan-II reduced ethanol drinking by signalling through the MC4R and suggested that this association is independent of food intake. Therefore our results of the association between the *MC4R* polymorphism and alcohol intake are novel in humans and mechanistically supported by results in mice [60]–[63]. With regard to *FTO* some studies have analyzed the influence of alcohol intake in humans with varying results [64], [65]. Among them we could mention a study carried out in Poland [65] that, in agreement with our results, also found a lower alcohol intake in carriers of the variant allele of the *FTO* rs9939609 polymorphism [49].

One limitation in our study may be that we have used self-reported measures of diet and physical activity in place of 24-hour record of food intake for diet pedometers or accelerometers that are more objective measures. However, taking into account our large sample size this was not possible due to the high cost that this would involve. Moreover, we have specifically validated the semi-quantitative 137-item food frequency questionnaire [43] as well as the MedDiet adherence questionnaire in this specific population obtaining excellent results [46]. On the other hand, the Minnesota Leisure Time Physical Activity Questionnaire used to measure physical activity as also validated in men [47] and women [48] from the Spanish population.

In conclusion, we have confirmed the association of the *FTO* rs9939609 polymorphism with higher BMI, waist circumference and obesity even in a high cardiovascular risk population and described an additive effect of the *FTO* rs9939609 and the *MC4R* rs17782313 on these obesity-related measures. Moreover, we have found that these genetic effects can be modulated by lifestyle variables, the statistical interaction with physical activity and the biological interaction (not statistically significant) with the adherence to the MedDiet being especially important in magnitude. Although in this population the *FTO* and the *MC4R* polymorphisms do not have a relevant association with food intake, their association with alcohol consumption is outstanding. This is the first time that an association of both polymorphisms with alcohol intake in humans is reported, although there are important data supporting the influence of the *MC4R* in alcohol intake in animal models. More studies are needed to replicate and extend these findings.

## Supporting Information

Figure S1 **Statistical interaction between the** ***FTO*** **rs9939609 polymorphism and physical activity (PA) in determining BMI (A) or waist circumference (B).** Adjusted means (n = 7,052) of BMI or waist circumference depending on the *FTO* genotypes according to the strata of physical activity (below and above 230 kcal/d). Means were adjusted for sex, age, center, diabetes and total energy intake. P values for the interaction terms were multivariate adjusted. In the stratified analysis by physical activity, P values for mean comparisons between genotypes in the low (P_1_) and high strata (P_2_) were multivariate adjusted. Error bars: SE of means.
(PDF)

Figure S2 **Interaction between the** ***FTO*** **rs9939609 and adherence to the Mediterranean Diet (AdMedDiet) on BMI (A) or waist circumference (B).** Adjusted means (n = 7,052) of BMI or waist circumference according to the strata of AdMedDiet (below and above 9 points). Means were adjusted for sex, age, field center, diabetes, total energy intake and physical activity. P values for the interaction terms and for mean comparisons between homozygous subjects for the variant allele were multivariate adjusted. Error bars: SE of means.
(PDF)

Table S1 **Association between the** ***FTO*** **rs9939609 and** ***MC4R*** **rs17782313 polymorphisms and food and alcohol intake in the studied population.**
(PDF)
