# Supplementary figures and images for: Statistical and Biological Gene-Lifestyle Interactions of MC4R and FTO with Diet and Physical Activity on Obesity: New Effects on Alcohol Consumption
Source: PLoS One. 2012 Dec 21;7(12):e52344. doi: 10.1371/journal.pone.0052344 (PMC3528751; doi:10.1371/journal.pone.0052344)

A

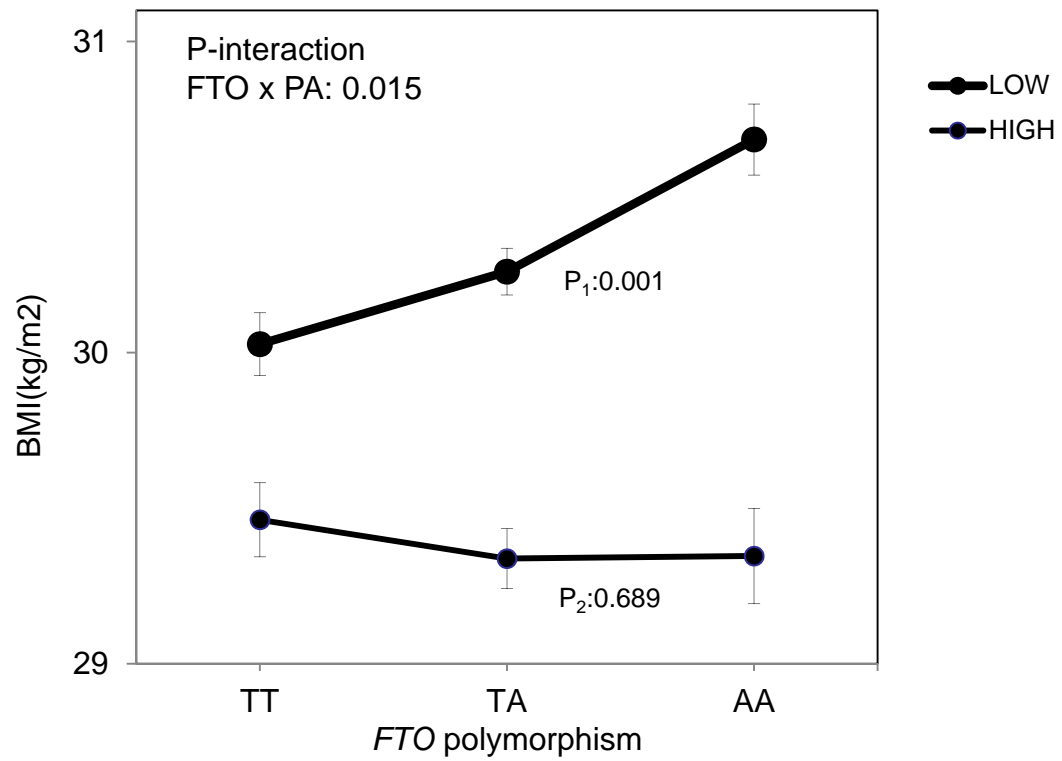

B

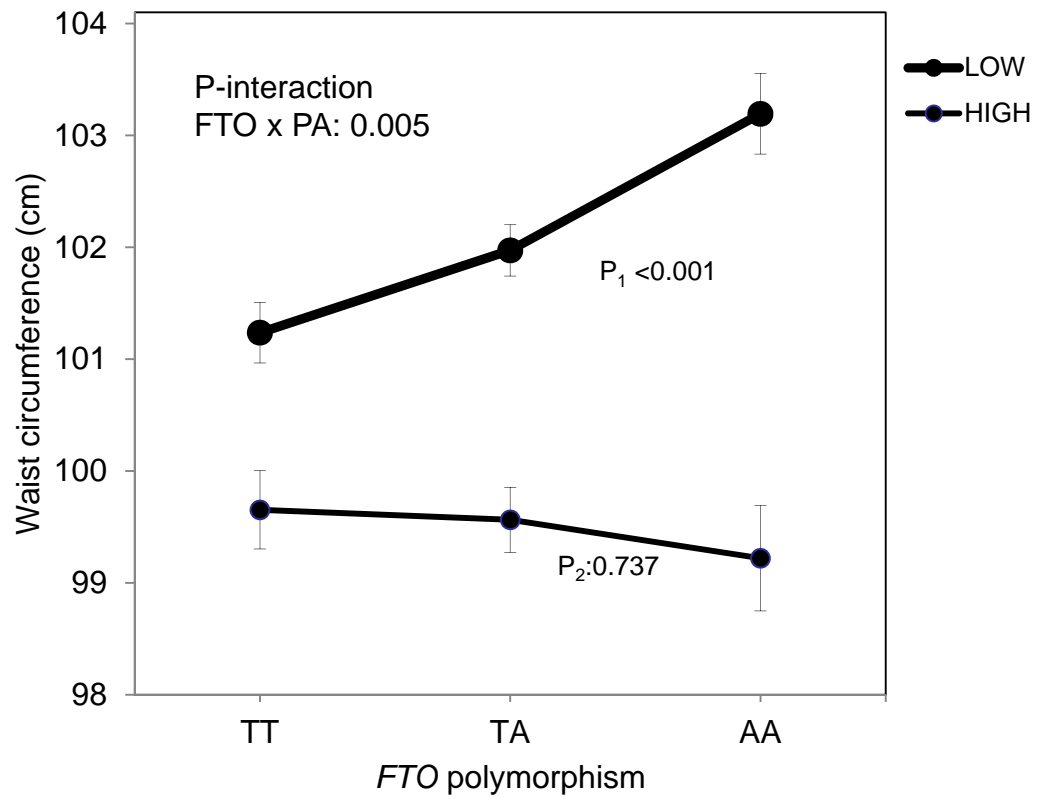

Supplement: Figure S1 — Statistical interaction between the FTO rs9939609 polymorphism and physical activity (PA) in determining BMI (A) or waist circumference (B). Adjusted means (n = 7,052) of BMI or waist circumference depending on the FTO genotypes according to the strata of physical activity (below and above 230 kcal/d). Means were adjusted for sex, age, center, diabetes and total energy intake. P values for the interaction terms were multivariate adjusted. In the stratified analysis by physical activity, P values for mean comparisons between genotypes in the low (P1) and high strata (P2) were multivariate adjusted. Error bars: SE of means. (PDF) [file pone.0052344.s001.pdf]

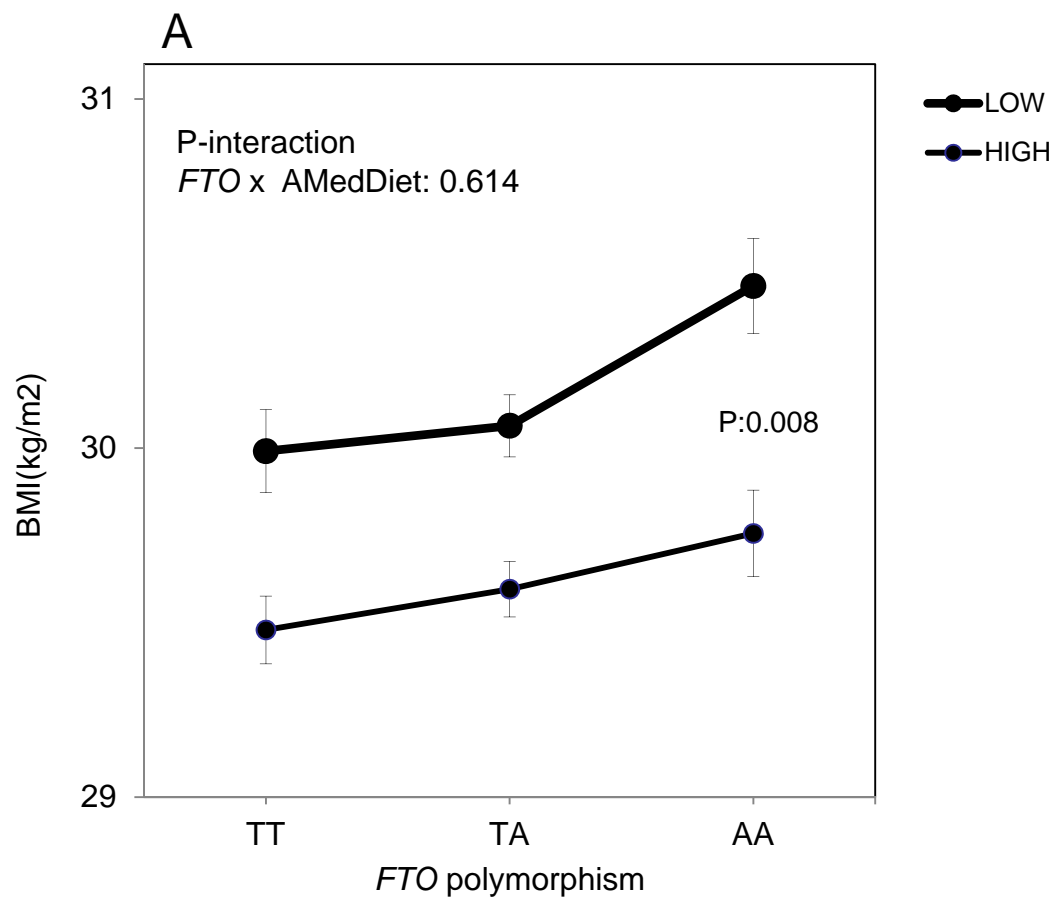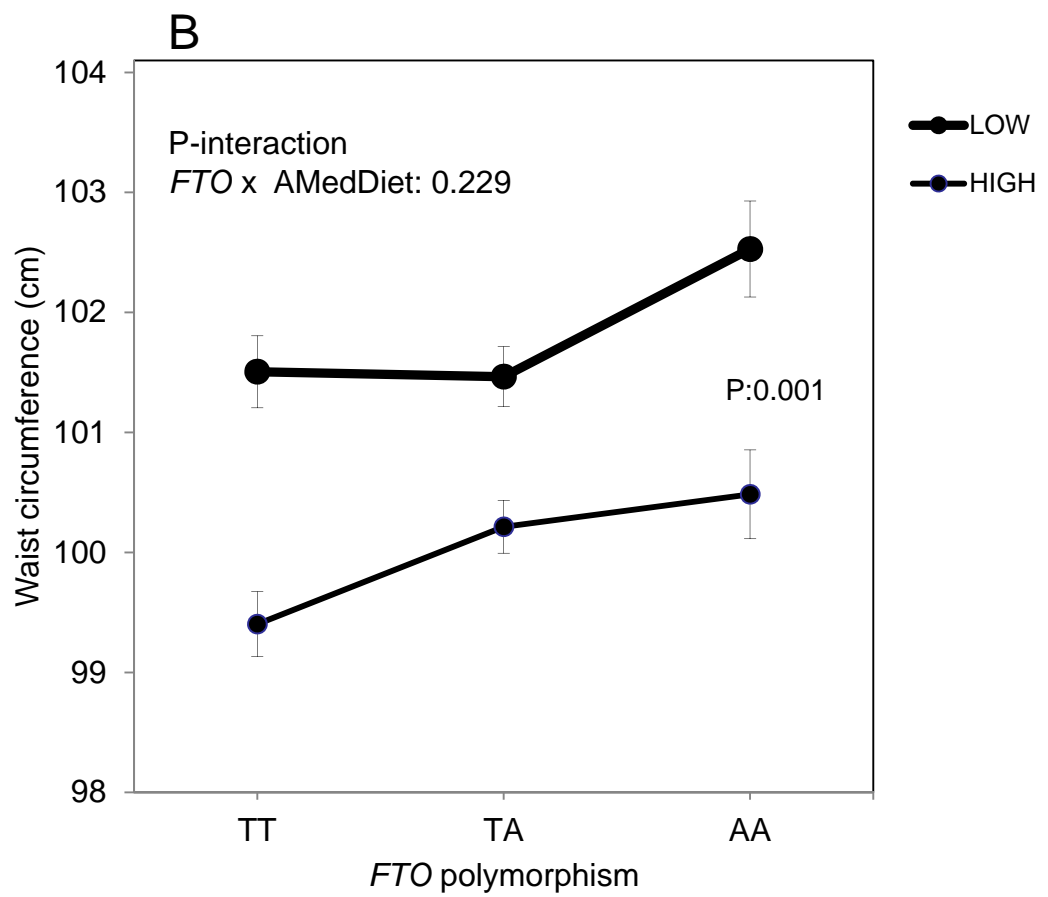

Supplement: Figure S2 — Interaction between the FTO rs9939609 and adherence to the Mediterranean Diet (AdMedDiet) on BMI (A) or waist circumference (B). Adjusted means (n = 7,052) of BMI or waist circumference according to the strata of AdMedDiet (below and above 9 points). Means were adjusted for sex, age, field center, diabetes, total energy intake and physical activity. P values for the interaction terms and for mean comparisons between homozygous subjects for the variant allele were multivariate adjusted. Error bars: SE of means. (PDF) [file pone.0052344.s002.pdf]
