# Supplementary material for: Statistical and Biological Gene-Lifestyle Interactions of MC4R and FTO with Diet and Physical Activity on Obesity: New Effects on Alcohol Consumption
Source: PLoS One. 2012 Dec 21;7(12):e52344. doi: 10.1371/journal.pone.0052344 (PMC3528751; doi:10.1371/journal.pone.0052344)
Supplement: Table S1 — Association between the FTO rs9939609 and MC4R rs17782313 polymorphisms and food and alcohol intake in the studied population. (PDF) [file pone.0052344.s003.pdf]

**Table S1: Association between the *FTO* rs9939609 and *MC4R* rs17782313 polymorphisms and food and alcohol intake in the Mediterranean population**

| | FTO (n=7,052) | | | | | | | Adjusted | | MC4R (n=7,019) | | | | | | | Adjusted | |
|---|---|---|---|---|---|---|---|---|---|---|---|---|---|---|---|---|---|---|
| | TT | | TA | | AA | | $P^1$ | B (95% CI) | $P^2$ | CC | | CT | | TT | | $P^1$ | B (95% CI) | $P^2$ |
| | Mean | SD | Mean | SD | Mean | SD | | | | Mean | SD | Mean | SD | Mean | SD | | | |
| Energy intake, kcal/*d* | 2288.2 | (616.1) | 2277.3 | (606.4) | ##### | (593.9) | 0.208 | -18.63 (-37.65, 0.39) | 0.055 | 2274.3 | (607.1) | 2279.2 | (611.4) | 2309.4 | (593.0) | 0.596 | 11.74 (-11.45, 34.40) | 0.327 |
| Total fat, *g/d* | 98.6 | (30.4) | 99.4 | (30.9) | 97.8 | (29.7) | 0.265 | -0.39 (-1.33, 0.54) | 0.405 | 98.7 | (30.7) | 98.9 | (30.7) | 101.3 | (30.2) | 0.340 | 0.46 (-0.66, 1.58) | 0.192 |
| SFA, *g/d* | 25.2 | (9.3) | 25.5 | (9.2) | 25.0 | (9.1) | 0.161 | -0.14 (-0.43, 0.16) | 0.359 | 25.3 | (9.3) | 25.3 | (9.1) | 26.3 | (9.4) | 0.137 | 0.22 (-0.14, 0.57) | 0.233 |
| MUFA, *g/d* | 48.9 | (16.1) | 49.1 | (16.2) | 48.4 | (15.7) | 0.381 | -0.24 (-0.72, 0.23) | 0.318 | 48.9 | (16.2) | 48.9 | (15.9) | 50.2 | (16.0) | 0.357 | 0.14 (-0.43, 0.72) | 0.334 |
| PUFA, *g/d* | 15.7 | (6.8) | 16.0 | (7.2) | 15.7 | (7.0) | 0.381 | 0.04 (-0.19, 0.27) | 0.725 | 15.9 | (7.0) | 15.9 | (7.0) | 16.0 | (6.9) | 0.890 | 0.05 (-0.23, 0.32) | 0.692 |
| Proteins, *g/d* | 92.8 | (23.4) | 92.9 | (23.4) | 91.8 | (22.6) | 0.314 | -0.57 (-1.32, 0.19) | 0.144 | 92.4 | (23.2) | 93.2 | (23.6) | 93.7 | (22.4) | 0.310 | 0.82 (-0.10, 1.73) | 0.079 |
| Carbohydrates, *g/d* | 242.0 | (83.1) | 238.2 | (80.1) | 237.3 | (80.1) | 0.136 | -2.34 (-4.90, 0.25) | 0.065 | 239.0 | (80.3) | 240.0 | (83.4) | 243.2 | (78.0) | 0.618 | 1.99 (-1.13, 5.11) | 0.198 |
| Alcohol, g/d | 8.8 | (14.7) | 8.3 | (13.9) | 7.7 | (13.8) | 0.113 | -0.49 (-0.91, -0.07) | **0.021** | 8.7 | (14.7) | 8.0 | (13.3) | 7.2 | (12.2) | 0.067 | -0.62 (-1.12, -0.11) | **0.018** |
| Wine, g/d | 61.5 | (113.2) | 56.4 | (104.6) | 52.6 | (99.6) | **0.043** | -4.06 (-7.28, -0.84) | **0.014** | 58.8 | (110.2) | 55.5 | (101.0) | 50.0 | (94.7) | 0.219 | -3.38 (-7.24, 0.50) | 0.090 |
| Beer, g/d | 45.3 | (143.1) | 44.8 | (131.9) | 38.0 | (124.7) | 0.240 | -4.18 (-8.50, 0.14) | **0.058** | 47.2 | (145.6) | 39.5 | (114.9) | 36.4 | (124.5) | **0.049** | -6.52 (-11.76, -1.29) | **0.016** |
| Adherence to MedDiet* | 8.6 | (2.0) | 8.7 | (2.0) | 8.7 | (1.9) | 0.893 | 0.02 (-0.04, 0.08) | 0.534 | 8.7 | (2.0) | 8.7 | (2.0) | 8.6 | (1.9) | 0.689 | -0.02 (-0.10, 0.05) | 0.641 |
| Vegetales, g/d | 337.8 | (161.4) | 338.1 | (144.0) | 339.0 | (147.6) | 0.975 | -0.34 (-5.13, 4.44) | 0.880 | 338.8 | (152.2) | 337.4 | (149.0) | 341.5 | (142.9) | 0.875 | 1.00 (-4.77, 6.77) | 0.702 |
| Fruits, g/d | 372.2 | (212.7) | 369.2 | (206.5) | 377.1 | (203.3) | 0.509 | 2.52 (-4.07, 9.12) | 0.764 | 370.4 | (205.1) | 375.5 | (213.2) | 361.4 | (207.3) | 0.411 | 2.53 (-5.42, 10.46) | 0.779 |
| Legumes, g/d | 20.8 | (14.8) | 20.9 | (13.6) | 20.5 | (12.5) | 0.617 | -0.17 (-0.60, 0.26) | 0.435 | 20.9 | (14.4) | 20.7 | (13.1) | 19.9 | (10.7) | 0.413 | -0.25 (-0.77, 0.28) | 0.365 |
| Cereals, g/d | 149.4 | (92.6) | 145.0 | (87.5) | 143.8 | (89.6) | 0.102 | -2.55 (-5.46, 0.36) | 0.182 | 145.7 | (90.1) | 147.5 | (89.7) | 147.8 | (84.6) | 0.698 | 1.94 (-1.56, 5.45) | 0.266 |
| Dairy products, g/d | 379.8 | (227.3) | 388.8 | (226.0) | 377.8 | (209.3) | 0.189 | -0.67 (-7.86, 6.50) | 0.855 | 383.9 | (222.0) | 382.7 | (223.1) | 399.2 | (239.3) | 0.451 | 2.62 (-6.03, 11.26) | 0.539 |
| Meats, g/d | 133.8 | (62.2) | 132.4 | (59.5) | 131.3 | (56.5) | 0.452 | -1.57 (-3.47, 0.32) | 0.104 | 132.1 | (61.4) | 133.7 | (57.5) | 133.2 | (57.1) | 0.592 | 1.14 (-1.15, 3.43) | 0.360 |
| Fish, g/d | 100.6 | (48.1) | 99.9 | (52.2) | 99.9 | (56.8) | 0.871 | -0.44 (-2.10, 1.23) | 0.608 | 99.3 | (48.8) | 101.1 | (56.9) | 101.5 | (52.7) | 0.364 | 1.59 (-0.41, 3.60) | 0.116 |
| Pastries, g/d | 23.1 | (32.1) | 22.3 | (29.8) | 22.2 | (28.9) | 0.563 | -0.51 (-1.51, 0.49) | 0.318 | 22.5 | (30.0) | 22.9 | (31.2) | 22.1 | (29.4) | 0.830 | -0.11 (-1.10, 1.31) | 0.883 |
| Olive oil, g/d | 39.7 | (18.2) | 39.3 | (18.0) | 38.9 | (17.9) | 0.418 | -0.31 (-0.85, 0.22) | 0.252 | 39.4 | (18.0) | 39.1 | (18.1) | 39.8 | (18.7) | 0.710 | -0.22 (-0.87, 0.43) | 0.308 |
| Nuts, g/d | 10.2 | (13.7) | 10.9 | (14.7) | 9.9 | (13.2) | 0.061 | 0.07 (-0.40, 0.53) | 0.784 | 10.5 | (14.4) | 10.4 | (13.7) | 10.7 | (13.7) | 0.937 | -0.04 (-0.57, 0.56) | 0.891 |

P1: Unadjusted P value for the comparison of means among genotypes.
P2: P value adjusted for sex, age, field center, diabetes and physical activity
